# Using Deep Learning to Perform Automatic Quantitative Measurement of Masseter and Tongue Muscles in Persons With Dementia: Cross-Sectional Study

**DOI:** 10.2196/63686

**Published:** 2025-03-19

**Authors:** Mahdi Imani, Miguel G Borda, Sara Vogrin, Erik Meijering, Dag Aarsland, Gustavo Duque

**Affiliations:** 1 Department of Medicine, Melbourne Medical School, University of Melbourne, St. Albans, Australia; 2 Centre for Age-Related Medicine (SESAM), Stavanger University Hospital, Stavanger, Norway; 3 Department of Neurology, Clínica Universidad de Navarra, Pamplona, Spain; 4 School of Computer Science and Engineering, UNSW Sydney, Sydney, Australia; 5 Department of Old Age Psychiatry, Institute of Psychiatry, Psychology, and Neuroscience, King's College London, London, United Kingdom; 6 Bone, Muscle & Geroscience Group, Research Institute of the McGill University Health Centre, McGill University, 1001 Decarie Blvd, Room EM1.3226, Montreal, QC, H4A 3J1, Canada, 1 514 934 1934

**Keywords:** artificial intelligence, machine learning, sarcopenia, dementia, masseter muscle, tongue muscle, deep learning, head, tongue, face, magnetic resonance imaging, MRI, image, imaging, muscle, muscles, neural network, aging, gerontology, older adults, geriatrics, older adult health

## Abstract

**Background:**

Sarcopenia (loss of muscle mass and strength) increases adverse outcomes risk and contributes to cognitive decline in older adults. Accurate methods to quantify muscle mass and predict adverse outcomes, particularly in older persons with dementia, are still lacking.

**Objective:**

This study’s main objective was to assess the feasibility of using deep learning techniques for segmentation and quantification of musculoskeletal tissues in magnetic resonance imaging (MRI) scans of the head in patients with neurocognitive disorders. This study aimed to pave the way for using automated techniques for opportunistic detection of sarcopenia in patients with neurocognitive disorder.

**Methods:**

In a cross-sectional analysis of 53 participants, we used 7 U-Net-like deep learning models to segment 5 different tissues in head MRI images and used the Dice similarity coefficient and average symmetric surface distance as main assessment techniques to compare results. We also analyzed the relationship between BMI and muscle and fat volumes.

**Results:**

Our framework accurately quantified masseter and subcutaneous fat on the left and right sides of the head and tongue muscle (mean Dice similarity coefficient 92.4%). A significant correlation exists between the area and volume of tongue muscle, left masseter muscle, and BMI.

**Conclusions:**

Our study demonstrates the successful application of a deep learning model to quantify muscle volumes in head MRI in patients with neurocognitive disorders. This is a promising first step toward clinically applicable artificial intelligence and deep learning methods for estimating masseter and tongue muscle and predicting adverse outcomes in this population.

## Introduction

Age-related muscle wasting and neurodegeneration, clinically presented as sarcopenia and dementia, respectively, are the major drivers of frailty, falls, and disability in older adults worldwide [1]. Sarcopenia is characterized by loss of muscle mass, strength, and function in older adults. Aging is the leading risk factor, but conditions such as chronic diseases, inflammation, sedentarism, and malnutrition promote sarcopenia onset and progression [2]. Sarcopenia has a 10% overall prevalence globally in older persons, 29% in the community, 14%-33% in long-term care settings, and up to 50% in the very old (>80) [3,4]. Despite being a common and relevant health-related condition, it is unseen and underdiagnosed, particularly in older persons with cognitive disorders. To diagnose sarcopenia, measurement of muscle mass, muscle performance, and strength is necessary [2]. Estimating muscle performance and strength is accessible and cheap with traditional methods, such as gait speed and grip strength, respectively [5]. However, techniques such as dual X-ray absorptiometry or body magnetic resonance imaging (MRI) are necessary to accurately assess lean or muscle mass. These methods can increase costs and time and are impractical in settings such as dementia clinics [6].

Dementia patients are highly affected by sarcopenia, with a prevalence of around 60%‐70% [7,8]. People living with neurodegenerative diseases are more prone to experience difficulties due to malnutrition, being sedentary, and falling; therefore, having sarcopenia increases the risk of adverse outcomes. Sarcopenia is not only a risk factor for adverse outcomes for those with dementia but also promotes cognitive loss in healthy older adults [9]. Therefore, diagnosing sarcopenia in people with neurodegenerative diseases is relevant and necessary.

Head MRI is a widely used diagnostic method for assessing dementia and Alzheimer disease (AD), as it offers intricate representations of the brain’s anatomy and physiology. In clinical practice, MRI is often combined with other imaging techniques and cognitive assessments to support the diagnosis of these conditions. The utilization of MRI has seen an upward trend in recent times, as it has become an instrumental tool for the early detection and tracking of the evolution of dementia and AD. According to estimates, 60%‐80% of patients diagnosed with dementia or AD undergo MRI as part of their diagnostic evaluation [6].

Mastication and deglutition muscles such as the masseter and tongue are visible in brain MRI scans [10,11]. These muscles can reflect not only age-associated general muscle decline but also systemic processes due to highly complex interactions with the immune system and the inflammatory response, the nervous system, and the crossroads of several components of the frailty syndrome [12,13]. Indeed, in a previous publication, we have reported that manually segmented masseter predicts mortality and clinical short-term and long-term outcomes in several contexts [14]. Moreover, head muscles such as the tongue and masseter could be cost-effective alternatives to estimate muscle mass in dementia and other common conditions such as head cancer, stroke, or cranioencephalic trauma [14,15]. However, manual and semi-automatic techniques are labor-intensive and time-consuming, making the image processing task for large studies difficult, expensive, and, most importantly, impractical to apply in a clinical setting. Therefore, in the present study, we aimed to use MRI scans of the head opportunistically to develop an automated deep learning method to evaluate sarcopenia.

## Methods

### Population and Data Source

The “Dementia Study of Western Norway” (DemVest) is a long-term study between 2005 and 2013, with ongoing follow-up assessments. Participants were referred from dementia clinics in Hordaland and Rogaland and were insured by the same national insurance scheme. The study’s methodology is described elsewhere [15]. Those with moderate or severe dementia, delirium, past bipolar or psychotic conditions, terminal illness, or newly diagnosed somatic diseases impacting cognition, function, or participation were excluded.

For this study, subjects with dementia with Lewy bodies (DLB) or mild AD who had baseline MRI scans were included. Out of 111 participants (85 AD and 26 DLB), 33 AD and 20 DLB participants with MRI images for brain and muscle measurement were selected based on the quality of the images and clear delineation of the regions of interest. The diagnosis of dementia was made in accordance with the Diagnostic and Statistical Manual of Mental Disorders, Fourth Edition (DSM-IV) criteria, and patients were classified as AD or DLB [16]. A mini-mental state examination score of ≥20 or a clinical dementia rating global score of 1 was chosen as the definition for mild dementia. The diagnosis was based on inclusion but could be modified with clinical evolution, consensus, and autopsy [15]. Participants were evaluated through structured assessments, and medical records were used to gather complete medical history and comorbidity data. In total, 56 participants had pathological diagnoses with 80% accuracy compared to clinical criteria, which reflects a reliable initial clinical diagnosis [17].

#### Ethical Considerations

This study was approved by the regional ethics committee (approval code: 2010/633) and the Norwegian authorities for the collection of medical data. All data were handled and kept following national data privacy protocols. All participants signed an informed consent form before inclusion in the study.

### Imaging

All images were acquired at baseline. A 1.5-T Philips Intera-scanner was used to obtain MRI images. The acquisition protocol for 3D T1-weighted sequence was as follows: flip angle of 30°, repetition time/echo time of 10.0/4.6 ms, number of excitations of 2, 2-mm slice thickness with 1-mm spacing between the slices (1-mm slices with no gap), matrix of 256×256 pixels, and field of view of 26 cm. Those with movement artifacts and inadequate image quality were removed from the data using visual quality checks. A standardized preprocessing method for harmonizing multiple collections of MRIs was applied, which consisted of movement correction and intensity normalization following previously validated techniques [15].

### Ground Truth Image Segmentation

**Figure 1.**
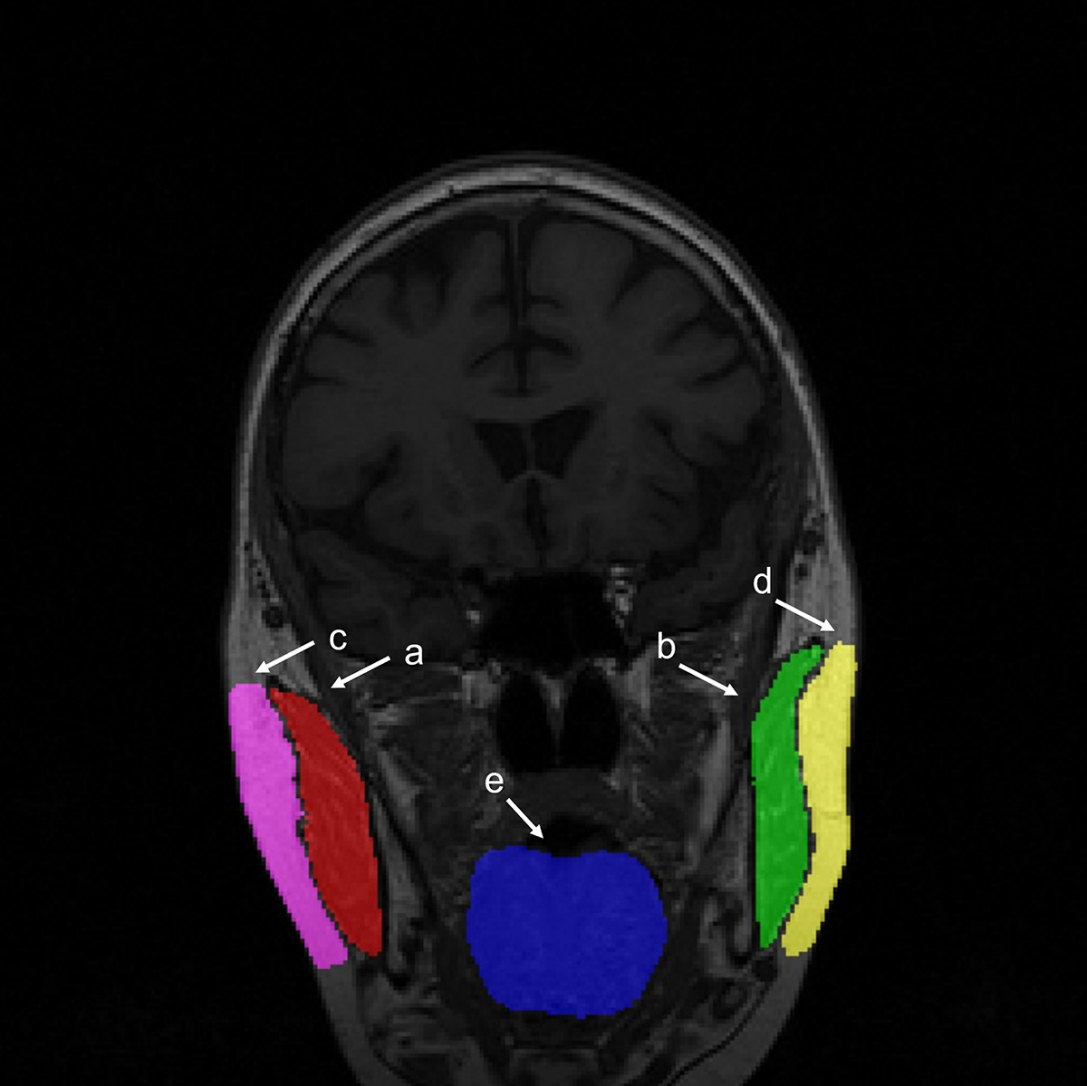
Example of segmented tissues overlaid on the original MRI. (**A**) Right masseter muscle, (**B**) left masseter muscle, (**C**) right subcutaneous fat, (**D**) left subcutaneous fat, and (**E**) tongue muscle.

Ground truth (GT) images were segmented using interactive pixel techniques made available by SliceOmatic software (TomoVision) following a manual method previously reported [18]. The size of the masseter muscle was used as a reference for the selection criteria of the slices. In total, 5 slices were selected from the ones with both right and left masseter muscles at their largest. For each slice, 5 tissues were segmented: left and right masseter muscles, left and right subcutaneous fat, and tongue muscle (Figure 1). The masseter muscle on each side was used as a reference to segment subcutaneous fat.

### Network Architecture

We studied and compared 6 different U-Net-like architectures. The original U-Net architecture [19] was first designed to segment medical images, and many other researchers have tried to improve its performance by integrating additional techniques into its architecture [20]. U-Net consists of a contracting path (encoder) and an expansive path (decoder) with skip connections between these 2 paths. The network learns features from the provided image and the mask at increasingly higher spatial scales by gradually down-sampling to lower resolutions through the encoding path. The expansive path then gradually increases the resolution of the output from the encoding path to the original image size, resulting in a probability map as an output, indicating the chance of each pixel belonging to a specific tissue. One important feature of U-Net is its skip connections, which concatenate feature maps from the encoding path to the corresponding block in the expansive path, making it possible to maintain small details crucial in medical image segmentation.

We have included 5 variants of U-Net in this study, including Attention U-Net, Dense U-Net, Residual U-Net, Inception U-Net, and U-Net++. Attention U-Net is desirable since it allows the model to focus on specific objects and ignore unnecessary areas [21]. In Dense U-Net, the traditional U-Net blocks are replaced with a dense block, enabling the model to segment objects with greater distinction. This feature is important in medical practice since tissues are often very close and sometimes overlap [22]. Residual U-Net architecture tries to tackle the vanishing gradient issue, a common problem in designing deep neural networks [23]. In most cases, the same organ’s size can vary between patients, which can cause limitations on the segmentation capability of the model. By using filters with different sizes, Inception U-Net attempts to overcome this problem [24]. Lastly, U-Net++ aids the classic U-Net model to more accurately segment images by providing semantic information from a dense network of skip connections as an intermediary grid between the encoding and decoding paths [25]. We also included a Wide U-Net architecture to eliminate the effect of increased trainable parameters. This model has the same architecture as U-Net but with more feature maps per layer (30, 60, 120, 240, and 480) compared to the original U-Net (16, 32, 64, 128, and 256). Hence, it will serve as a control to compare the models with larger numbers of trainable parameters with the base U-Net (Table 1).

**Table 1.** Number of trainable parameters for each model.

Model	Trainable parameters
U-Net	2,164,390
Attention U-Net	2,233,270
U-Net++	2,555,702
Inception U-Net	5,529,526
Residual U-Net	6,877,110
Dense U-Net	7,666,320
Wide U-Net	7,596,306

### Training Procedure

All models were trained with the mini-batch stochastic gradient descent algorithm using the Adam optimizer. A batch size of 8 was selected. The learning rate was 0.0001. The training was done for 200 epochs. The values of hyperparameters were empirically tuned for best performance. Categorical cross-entropy (CSE) was selected as the loss function for this study [26]. The CSE loss function minimizes the distance between 2 distributions (the predicted labels and the GT labels). CSE is one of the most popular loss functions for image segmentation and has shown excellent performance in muscle segmentation [27,28]. All experiments were implemented with open-source software: Python (version 3.7.13), TensorFlow (version 2.8.2), and Keras (version 2.8.0).

### Model Evaluation

The results of the experiments were evaluated using 2 main measures: Dice similarity coefficient (DSC) and average symmetric surface distance (ASSD). The DSC represents the agreement between the GT labels and predicted labels that models generate:


$$\text{DSC}\left(P,G\right)=\frac{2(P\cap G)}{\left(P+G\right)}$$

where ∩ is the intersection and P and G are the 2 labels. DSC ranges between 0 and 1, where 0 indicates no agreement and 1 indicates perfect agreement. In our study, DSC is presented as a percentage.

The ASSD measures the average distance from pixels on the boundary of predicted labels to corresponding pixels on the boundary of the GT labels:

where $B_{P}$ and $B_{G}$ are the boundaries of predicted labels and corresponding reference labels, respectively. d(v,B) is the shortest Euclidean distance between voxel v and boundary B. An ASSD of 0 indicates a perfect match between predicted and reference labels. The ASSD was measured in mm.

We used k-fold cross-validation in evaluating the models. This technique splits the data set into k subsets (folds). The deep learning models are trained on all but one of the subsets (k–1), and then the models are evaluated on the subset that was not used for training. This process is repeated k times, and the average of the results is reported. We used k=10.

Additional metrics, including Jaccard coefficient, precision, recall, sensitivity, specificity, and F_1_-score, are presented in Multimedia Appendix 1. The results are shown as mean and standard deviation and median and interquartile range across k-fold (k=10) cross-validation.

### Statistical Methods

We explored the association between the BMI and the muscle and fat areas and volumes in the MRI images using individual linear regression models adjusted by sex and age. No adjustments for multiple testing were made. All assumptions were checked. All *P*-values were evaluated at a 5% level. The analysis and graphs were carried out using R (version 4.2.2).

## Results

### Segmentation

**Figure 2.**
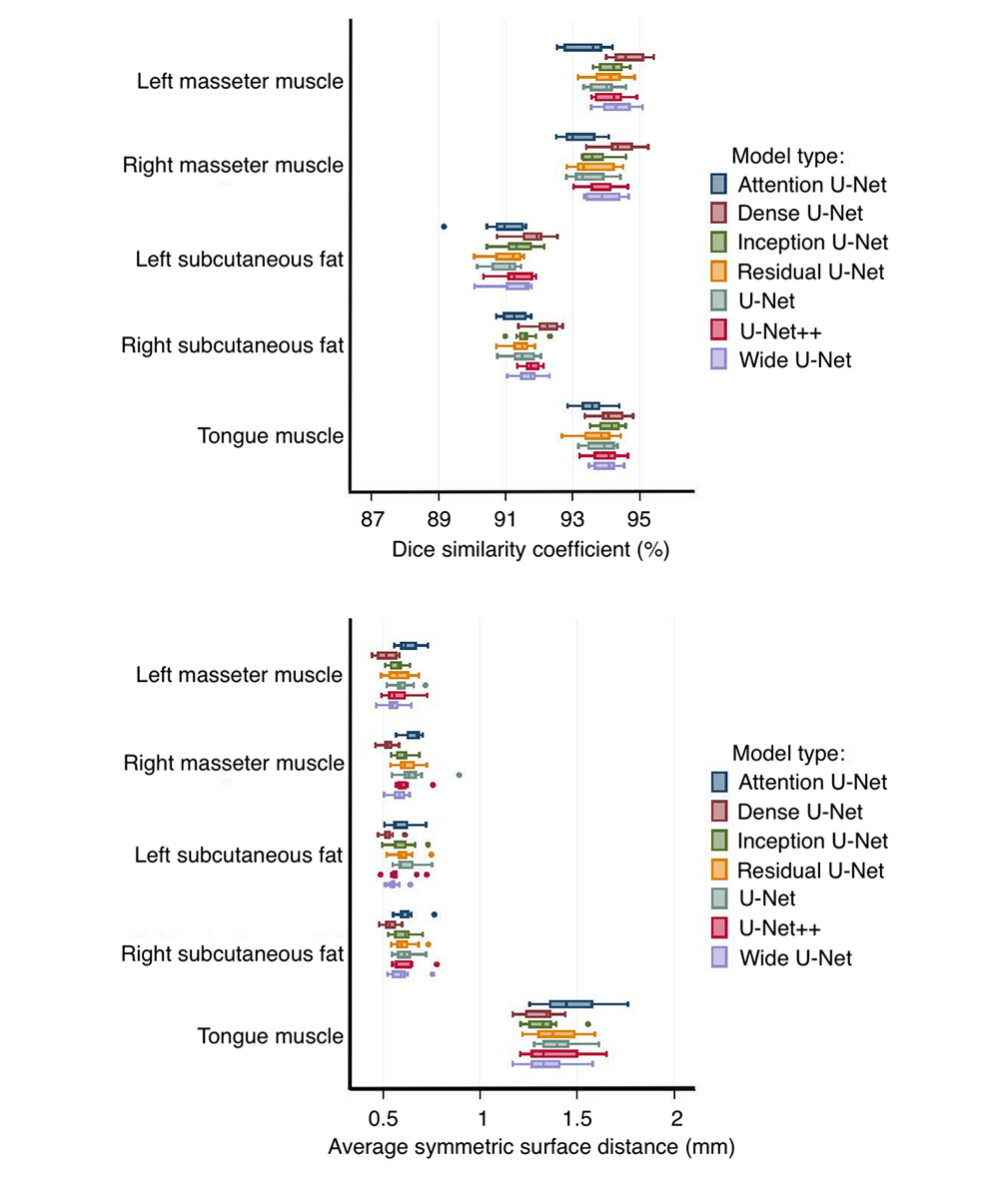
Box plot of k-fold (k=10) cross-validation results for attention U-Net, dense U-Net, inception U-Net, residual U-Net, U-Net, U-Net++, and wide U-Net. Top: DSC in percentage. Bottom: ASSD in mm.

DSC and ASSD were used to quantitatively analyze the segmentation results (Figure 2 and Table 2). The Dense U-Net model has a higher average DSC and lower ASSD than other models. In contrast, Attention U-Net has the lowest average DSC score compared to other models. This result is confirmed by the higher ASSD for Attention U-Net for all tissues. Other models have shown almost similar results for all areas. Additional metrics have been presented in Multimedia Appendix 1. The results from these metrics confirm the findings of the study mentioned in this section; hence, they were omitted from the main manuscript.

**Table 2.** Mean DSC and ASSD for test and validation results for k-fold (k=10) cross-validation. Standard deviation for the measurements in this table is presented in Multimedia Appendix 1.

	Model	Test set					Validation set				
		T^a^	LM^b^	RM^c^	LSF^d^	RSF^e^	T	LM	RM	LSF	RSF
DSC^f^(%)	Attention U-Net	93.57	93.4	93.19	90.9	91.26	93.43	93.38	93.23	91.05	91.43
	Dense U-Net	94.12	94.66	94.4	91.76	92.22	94.07	94.7	94.49	91.89	92.42
	Inception U-Net	94.07	94.12	93.69	91.36	91.59	93.85	94.27	93.73	91.45	91.81
	Residual U-Net	93.71	94.03	93.53	91.05	91.46	93.74	94.1	93.67	91.35	91.68
	U-Net	93.83	93.94	93.46	90.97	91.49	93.88	93.93	93.52	91.18	91.67
	Wide U-Net	94	94.29	93.93	91.35	91.68	94.03	94.3	93.99	91.59	92.01
ASSD^g^(mm)	Attention U-Net	1.47	0.63	0.66	0.59	0.62	1.53	0.67	0.65	0.6	0.59
	Dense U-Net	1.3	0.52	0.53	0.53	0.54	1.37	0.51	0.53	0.52	0.54
	Inception U-Net	1.33	0.57	0.61	0.59	0.61	1.44	0.56	0.63	0.6	0.59
	Residual U-Net	1.39	0.58	0.62	0.61	0.61	1.38	0.58	0.63	0.58	0.6
	U-Net	1.41	0.6	0.65	0.62	0.61	1.45	0.6	0.64	0.61	0.59
	Wide U-Net	1.35	0.55	0.58	0.56	0.6	1.34	0.55	0.58	0.56	0.57

a T: tongue muscle.

b LM: left masseter muscle.

c RM: right masseter muscle.

d LSF: left subcutaneous fat.

e RSF: right subcutaneous fat.

f DSC: Dice similarity coefficient.

g ASSD: average symmetric surface distance.

### Clinical Validation

**Figure 3.**
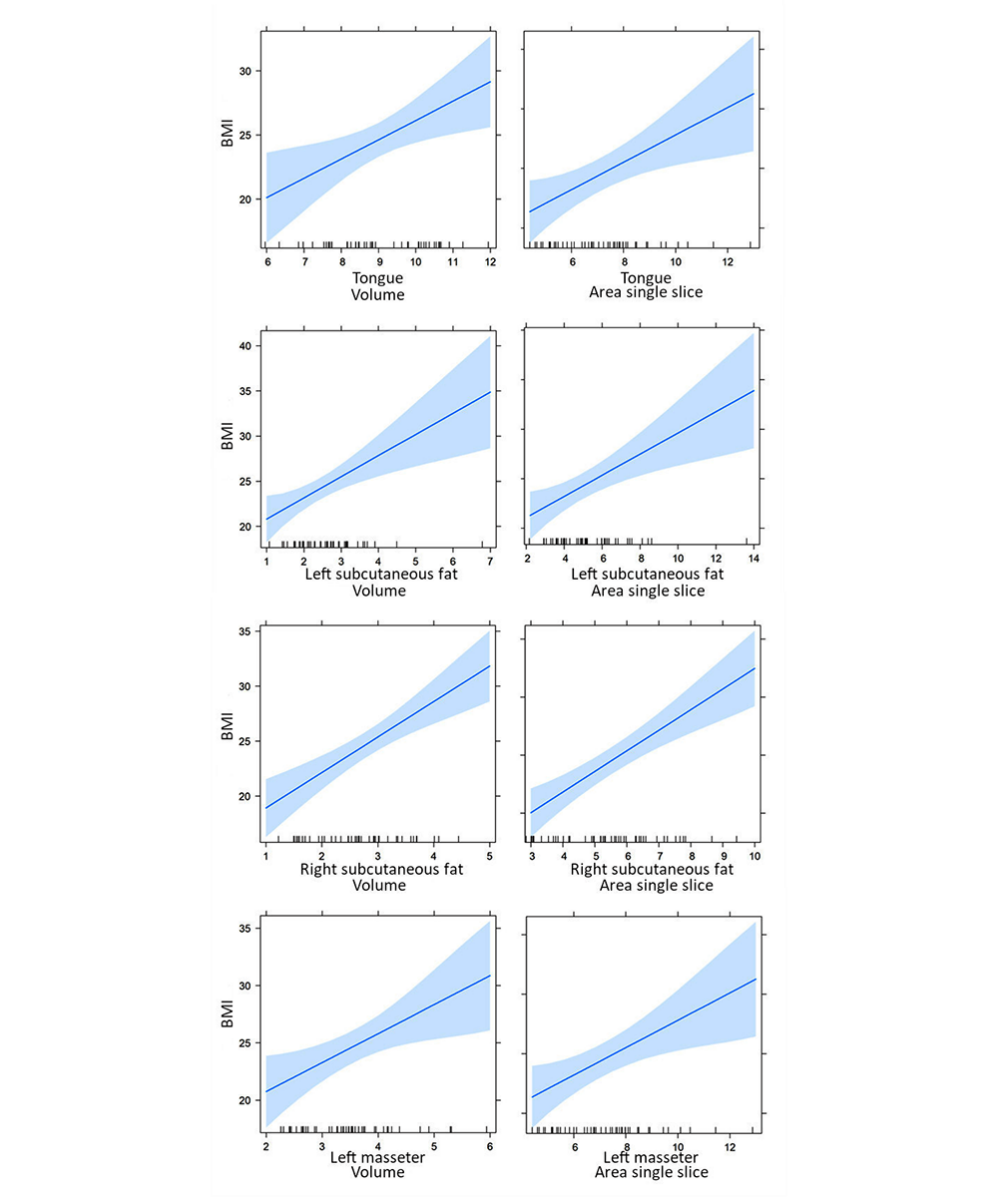
Individual linear regression repressing the relationship between quantitative results (area of a single slice and volume of 5 slices) from the Dense U-Net model and BMI.

To prove the clinical validity of the measurements, we evaluated the association between the segmented muscles and subcutaneous fat and BMI. We found a significant positive association between tongue muscle, left masseter muscle, and left and right subcutaneous fat and BMI (Figure 3). The area of a single slice as well as the volume of 5 slices per patient was calculated for this experiment. The results were adjusted by age and sex with *P*<.05 (Multimedia Appendix 2).

## Discussion

In this study, we evaluated the performance of six deep learning models for segmentation of the masseter muscles, subcutaneous fat, and tongue muscle in MRI images of the head. Several variations of the U-Net architecture were trained and tested using k-fold cross-validation. The use of deep neural networks for segmenting musculoskeletal tissues in patients with AD and LBD is a novel experimental contribution to the deep learning-based segmentation literature as well as the clinical literature.

Our study demonstrated that the Dense U-Net model performed superiorly to the other models in all evaluated regions by achieving an overall DSC of 93.43% and ASSD of 0.68 mm on the test data. The remaining models demonstrated comparable outcomes, except the Attention U-Net, which achieved slightly less accurate results in all regions with an overall DSC of 92.46% and ASSD of 0.79 mm on the test data. Notably, despite having a comparable number of trainable parameters, the Dense U-Net model demonstrated a higher DSC and lower ASSD than the Wide U-Net model. The Dense U-Net model achieves superior performance with a similar number of parameters, suggesting that its architectural efficiency, rather than parameter quantity alone, drives this improvement.

The validation set produced similar results to the training set, indicating that the trained models did not suffer from overfitting. Although we applied data augmentation to the training data set (results not shown), it did not significantly improve the accuracy of the segmentation models.

Furthermore, we observed a significant correlation between the results and BMI, a well-established measure of nutrition and body composition. This underscores the validity and clinical relevance of this method. If these localized muscle measurements correlate with BMI, it suggests that they may reflect broader nutritional and body composition states.

Sarcopenia is a relatively newly recognized condition for which neuromuscular degeneration, central nervous system alpha motor unit loss, and fat infiltration into muscle are the most distinctive proven and observed pathogenic features, leading to loss of muscle mass and strength and predisposing to physical frailty [2]. We have proposed that non-invasive assessment of intermuscular adipose tissue and muscle mass by image analysis could constitute a viable method to diagnose sarcopenia and predict its associated outcomes, the clinical impact of which is also under study by our group [29].

The validity and clinical implications of measuring the masseter muscle have been shown in previous studies [30,31]. In recent work by our group, we compared the diagnostic capacity for sarcopenia between the gold-standard dual X-ray absorptiometry and our measurements of head muscles. The results showed that both methods had equivalent accuracy [32]. In older adults with glioblastoma, a decreased masseter diameter on preoperative imaging was associated with shorter overall survival and 90-day mortality after surgical resection [33]. In addition, low masseter muscle was significantly associated with worse overall survival in patients aged 65 years or older, diagnosed with squamous cell carcinoma of the head and neck and treated with curative intent [34]. Another study evaluated post-operative results after carotid endarterectomy; low masseter mass was associated with a prolonged hospital stay and recurrent stroke within 5 years [35]. In another study, preoperative masseter mass was a predictor of postoperative pneumonia in patients with esophageal cancer [36]. Additionally, other studies have shown that the masseter muscle can be used as a nutritional biomarker. The masseter muscle, analyzed via computed tomography (CT) anthropometry, showed a statistically significant association with systemic nutritional biomarkers [37]. On the other hand, the tongue has shown to be a good marker of prognosis, as tongue strength has shown to be helpful in diagnosing sarcopenia [38]. Previous studies from our group also report that tongue muscle volume is correlated with malnutrition and even brain structures in patients with dementia [18,29].

Therefore, the approach we present in this paper can be opportunistically used to quantify muscle volumes and investigate the implications of having low muscle mass in the masseter or the tongue in people with brain, head, and neck diseases. Thus, it is an important first step toward developing a more efficient method to estimate masseter and tongue muscle with a better capacity to be implemented in clinical practice. Manual and semi-automatic techniques have been employed in several studies for masseter muscle segmentation in MRI [39]. A recent study used shape determination to segment the masseter muscle in MRI images [40]. In this approach, a manual contour for 8 slices must be defined by the user, and the model then determines the shape for the remaining scans, reducing the time and labor required for segmentation. However, this technique still requires manual segmentation, which can be time-consuming and prone to user error compared to our approach.

Model-based techniques have also been explored for the segmentation of the mastication muscle [41,42]. Although these techniques have shown high accuracy (>90%), there must be a distinct boundary between the masseter muscle and surrounding tissues to ensure accuracy. This distinct boundary refers to visible differences in intensity, texture, or anatomical structure on imaging, which enable the models to accurately separate the muscle from adjacent tissues, such as fat or bone.

Machine and deep learning approaches have been widely used to segment muscles in various body parts. CT scans and cone beam CT scans have primarily been investigated for measuring the head organs, including masseter muscle using these techniques [43-45]. In a previous study, a basic U-Net model was applied to segment the masseter muscle in head CT scans to investigate hemifacial microsomia [46]. The mean DSC reported in that study was 79.4% for the experimental group and 82.4% for the group with mandible deformities, which are lower compared to the results obtained in our study. In a study using CT scan of the head for segmentation of masticatory muscles, deep learning techniques were superior to atlas-based techniques, achieving a mean DSC of 83% [47]. To the best of our knowledge, the techniques considered in our study have not yet been used to segment musculoskeletal tissues in MRI images of the head.

Our study had some limitations. The Attention U-Net model demonstrated the lowest DSC values and the highest ASSD values among the evaluated models, indicating suboptimal segmentation accuracy across tissues. This underperformance may be attributed to the Attention mechanism’s inability to effectively focus on the target tissue, leading to dispersed attention, particularly in smaller structures or regions with indistinct boundaries. To address this limitation, future work could involve refining the Attention mechanism to enhance its specificity and focus on regions of interest. Alternatively, exploring models that prioritize multi-scale feature extraction and detail preservation may provide improved segmentation performance, particularly for small or complex anatomical regions.

The DemVest study had some limitations that may have impacted the results. Selection bias might have been present if patients with more complex health conditions were included, as primary care referrals were used. The study was not specifically designed for the paper’s purpose, which could limit the data analysis and interaction control. For example, the absence of a healthy control group prevents us from determining whether the observed muscle volume characteristics are specific to individuals with AD or neurocognitive disorders, or if they represent normal variations associated with aging. However, the primary objective of this study was to demonstrate the feasibility and accuracy of our deep learning model in quantifying muscle volumes using head MRI, rather than to establish definitive differences between diseased and healthy populations. In addition, the sample size is relatively small, and there is a risk that our results may not generalize to larger populations. Additionally, the MRI scans were obtained from a single center using a single MRI machine, which may affect the model’s generalizability. Longitudinal analyses were not conducted because imaging was only performed at baseline, in line with the initial primary objectives of the DemVest study and the resources allocated for image acquisition.

These limitations should be considered when interpreting this study’s results and addressed in future studies seeking broader application of the proposed approach. Whether masseter and tongue volumes in these muscles correlate with lean body mass and inflammaging and could be predictors of neurocognitive conditions and their associated outcomes remains unknown.

On the other hand, this study has several strengths, including well-characterized data, detailed and exhaustive diagnosis to correctly identify people with dementia, and an automated quality control and analysis pipeline. Additionally, the results fill a gap in the literature and provide insights into a possible method to efficiently diagnose sarcopenia in context when a head MRI is already available.

In summary, to our knowledge, this is the first study that validates deep learning methods that could be easily implemented in clinical practice to measure masseter and tongue muscle volumes with a solid potential to become biomarkers with strong predictive value for adverse outcomes in older persons with dementia. Since imaging is widely used in memory clinics worldwide, this opportunistic approach to image analyses could become standard practice in those settings. However, further large longitudinal studies are still required.

## Supplementary material

10.2196/63686 Multimedia Appendix 1 Table presenting extended accuracy evaluation metrics, including Dice Similarity Coefficient (DSC), Jaccard Coefficient (JC), Average Symmetric Surface Distance (ASSD), Precision, Recall, Sensitivity, Specificity, and F1-Score. Results are presented as mean (standard deviation) and median (interquartile range) across 10-fold cross-validation.

10.2196/63686 Multimedia Appendix 2 Table presenting association between area and volumes from MRI scans and BMI in each region of study.

## Abbreviations

AD: Alzheimer disease
ASSD: average symmetric surface distance
CSE: categorical cross-entropy
CT: computed tomography
DLB: dementia with Lewy bodies
DSC: Dice similarity coefficient
DSM-IV: Diagnostic and Statistical Manual of Mental Disorders, Fourth Edition
GT: ground truth
MRI: magnetic resonance imaging
