# Supplementary material for: Using Deep Learning to Perform Automatic Quantitative Measurement of Masseter and Tongue Muscles in Persons With Dementia: Cross-Sectional Study
Source: JMIR Aging. 2025 Mar 19;8:e63686. doi: 10.2196/63686 (PMC11999904; doi:10.2196/63686)
Supplement: Multimedia Appendix 2 [file aging-v8-e63686-s002.docx]

Supplementary Table 1 – Association between area and volumes from MRI scans and BMI in each region of study.

|  |  | **Adjusted models** | | |
| --- | --- | --- | --- | --- |
|  |  | **Estimate** | **Standard error** | **P-value** |
| **Area** | **Tongue muscle** | 0.587 | 0.255 | .02 |
|  | **Left masseter muscle** | 1.15 | 0.397 | .006 |
|  | **Left subcutaneous fat** | 1.067 | 0.323 | .002 |
|  | **Right masseter muscle** | 0.626 | 0.462 | .182 |
|  | **Right subcutaneous fat** | 1.774 | 0.341 | .000 |
| **Volume** | **Tongue muscle** | 1.504 | 0.54 | .008 |
|  | **Left masseter muscle** | 2.53 | 0.919 | .008 |
|  | **Left subcutaneous fat** | 2.342 | 0.687 | .001 |
|  | **Right masseter muscle** | 1.669 | 0.905 | .072 |
|  | **Right subcutaneous fat** | 3.231 | 0.667 | .000 |

P-value significant at < .05
